# Levels of organophosphorus pesticides in medicinal plants commonly consumed in Iran

**DOI:** 10.1186/2008-2231-20-9

**Published:** 2012-08-28

**Authors:** Parisa Sarkhail, Masud Yunesian, Reza Ahmadkhaniha, Pantea Sarkheil, Noushin Rastkari

**Affiliations:** 1 Pharmaceutical Sciences Research Center, Tehran University of Medical Sciences, Tehran, Iran; 2 Center for Air Pollution Research (CAPR), Institute for Environmental Research (IER), Tehran University of Medical Sciences, Tehran, Iran; 3 School of Public Health, Tehran University of Medical Sciences, Tehran, Iran

**Keywords:** Organophosphorus pesticides, Medicinal plants, HS-SPME-GC-MS, Borage

## Abstract

The frequent occurrence of pesticide residues in herbal materials was indicated by previous studies. In this study, the concentration of some of the organophosphorus pesticides including parathion, malathion, diazinon and pirimiphos methyl in different kinds of medicinal plants were determined. The samples were collected randomly from ten local markets of different areas of Iran. At the detection limit of 0.5 ng g^-1^, parathion and pirimiphos methyl were not detected in any of the samples_._ Some amounts of malathion and diazinon were found in *Zataria*, *Matricaria chamomile*, *Spearmint* and *Cumin* Seed samples while, the concentrations of target organophosphorus pesticides in *Borage* samples were below the detection limits of the methods which could be a result of intensive transformation of organophosphorus pesticides by *Borage*. In addition the organophosphorus pesticides were detected in all of the samples below the maximum residue levels (MRLs) proposed by the international organizations.

## Introduction

Medicinal plants are widely consumed as raw materials for pharmaceutical preparations and as supplements for dietetic products and especially for “self-medications” in the general population and they are also commonly used in health care products, food additives or supplementary foods [1]. Therefore, a large quantity of them is consumed in both daily life and pharmaceutical industry. In addition, medicinal plants are increasingly favored because of their distinct curative effects and naturally physiologic properties. However, pesticides are often used in order to improve productivity and profit margins in the production of medicinal plants. Hence pesticide residues in medicinal plants become pitfalls in safety and present obstacles to be acknowledged by the international community. Up to now, many international organizations and countries have set up regulations concerning the pesticides in the plants and plant products [2]. In recent years, with the significant improvements in pesticides analytical techniques and tremendous concerns in the safety of consumers’ products, the pesticides residues in foods have been more strictly monitored in the aspects such as classes and/or amounts as well as MRLs (maximum residue levels) in the developed countries [1-4]. The results of the works on pesticide residues in crude herbal materials indicate that the presence of pesticide residues is quite common [5-7]. In Iran, up till now, studies dealing with the detection of pesticide residues in spice and medicinal plants have been lacking. This study was conducted to have a view of the situation of medicinal herb pollution by organophosphorus pesticides (OPPs) in Iran. To achieve this aim the levels of frequently used OPPs in 50 samples of five kinds of medicinal plants which are consumed mostly in Iran was determined by a sensitive headspace solid-phase GC-MS method developed in the previous studies [8-10].

## Materials and methods

### Sampling

Ten samples of each group of plant, *Zataria* (*Zataria multiflora*), *Matricaria chamomile* (*Matricaria recutita*), *Borage* (*Borage officinalis*), *Spearmint* (*Mentha spicata*) and *Cumin Seed* (*Cuminum cyminum*) were collected randomly from ten local markets of different areas of Iran (Rasht, Sari, Mashhad, Tehran (2 markets), Semnan, Tabriz, Isfahan, Shiraz, Ahvaz) in March 2011. The plant materials were powdered, sieved (1–2 mm) and stored away from light and moisture in airtight containers until analysis (two months later).

### Analytical method

**Figure 1:**
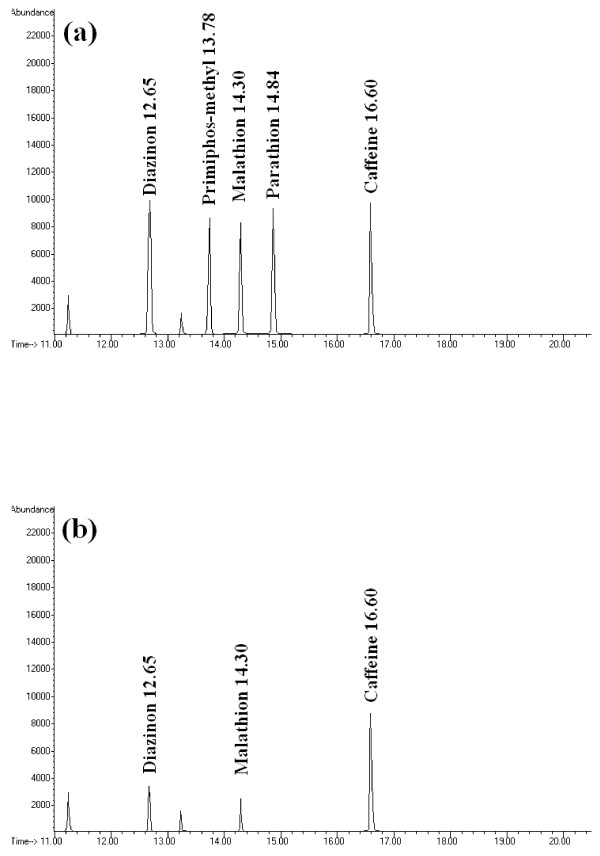
**Representative HS-SPME-GC-MS chromatogram (SIM mode) of (a) a quality control sample, spiked with OPPs and Caffeine at 30 ng g**^**-1**^**, and (b) one of the studied* Zataria *sample**.

The method used for the analysis was developed in our previous study [8]. The method is based on headspace solid-phase microextraction of target compounds followed by GC-MS for qualification and quantification. In brief, the OPPs were extracted from the plants samples by liquid-liquid extraction followed by preconcentration step using an in-house developed head space solid-phase microextraction (HS-SPME) fiber coated with single walled carbon nanotubes (SWCNTs). Then the analytes were determined by gas chromatography–mass spectrometry (SIM mode). The identification of pesticides was based on GC retention time, mass spectrum and comparison with authentic standards. A representative chromatogram of one of *Zataria* samples is shown in Figure 1.The optimum analytical parameters were determined by one factor at a time method as described elsewhere [10]. The analytical method was validated based on international guidelines [9]. To determine the method performance three different samples of each target plant which have not been treated with OPPs (wild species) were collected and considered as blank samples. The quality control (QC) samples were prepared at three levels by using these blank matrices spiked with authentic standards [8].

## Results

A total of 50 samples of different medicinal plants were analyzed (10 sample of each plant). Frequency and mean concentration (ng g^-1^) of pesticide in the analyzed samples are outlined in Table 1. At the detection limits of 0.5 ng g^-1^ parathion and pirimiphos methyl were not detected in any of the samples, whereas the other OPPs were found in some of the samples. Furthermore the results indicated that *ZatariaMatricaria chamomileSpearmint* and *Cumin* Seed had some detectable concentrations of at least one of the OPPs, while, *Borage* samples showed no detectable amounts of any of the OPPs. The results also indicated that malathion was the pesticide detected the most. Residues of both malathion and diazinon were detected together in some of the samples. The highest mean levels of malathion 24.2 ± 9.68 ng g^-1^ were detected in *Chamomile*, while the *Spearmint* samples showed the highest levels of diazinon (98.2 ± 14.53 ng g^-1^). The results indicated that in 15 samples (30% of total samples) no residue was found, 35 (70%) samples contained OPPs residue below MRL (maximum residue limit) established by either World Health Organization (WHO) or European Union (EU) [11]. Of the total samples, 3 (6%) samples contained both malathion and diazinon residues. The highest levels of malathion and diazinon residues were determined as 36 and 112 ng g^-1^ respectively.

**Table 1:** **Levels of malathion, diazinon, parathion and pirimiphos methylin studied medicinal plant samples (ng g**^**-1**^**)**

**Sample**	**Mean concentration ± (SD)**							
	**Malathion**	**No of sample (detected)**	**Diazinon**	**No of sample (detected)**	**Parathion**	**No. of sample (detected)**	**Pirimiphos methyl**	**No. of sample (detected)**
*Borage*	n.d	0	n.d	0	n.d.	0	n.d.	0
*Spearmint*	14.9 ± (3.66)	6	98.2 ± (14.53)	5	n.d.	0	n.d.	0
*Matricaria chamomile*	24.2 ± (9.68)	6	48.3 ± (6.33)	4	n.d.	0	n.d.	0
*Zataria*	5.0 ± (1.93)	6	10.2 ± (3.55)	2	n.d.	0	n.d.	0
*Cumin* Seed	4.1 ± (1.56)	5	7.6 ± (2.17)	3	n.d.	0	n.d.	0

Note: n.d., not detected.

## Discussion

The demand for medicinal herbs in recent years is significantly increasing. Many people are refraining from using synthetic pharmaceutical drugs and resorting to the safer, naturally occurring products. Consumers of medicinal plants are expecting these herbs to be products of good agriculture practices, with no residues of environmental pollutants especially pesticides. In practice, the large-scale cultivation of medicinal as well as food plants is not possible without pesticides. Because of high costs, ‘organic’ (pesticide-free) cultivation is only possible on a small scale or the risk of severe attack by pests must be accepted [12]. The use of OPPs helped increase productivity, but caused great concern about their effect on human health and safety. Previous studies indicated that pesticides exposure can cause both acute and chronic metabolic disorders such as diabetes. The underlying mechanisms including impairment of the enzymatic pathways involved in the metabolism of carbohydrates, fats and protein within cytoplasm, mitochondria, and peroxisomes could primarily be the result of Acetylcholine esterase inhibition, the main function of OPPs. Furthermore OPPs can induce cellular oxidative stress via affecting mitochondrial function and therefore disrupt neuronal and hormonal status of the body. Therefore many studies have been conducted to determine the exposure levels to pesticides and related health risk via food and beverage consumption [13]. The aim of the present research was to determine the residues of target OPPs in some of the medicinal plants to have a view of the situation of medicinal herb pollution by OPPs in Iran. The target OPPs were selected based on the report of Ministry of Agriculture of Iran about the most imported OPPs between 2005 and 2010 [14]. As the results indicated, in all the samples, residues of parathion and pirimiphos methyl were below the detection limits (<0.5 ng g^-1^). Among various OPPs in the present study, residues of malathion and diazinon are predominant. Diazinon and malathion are the only recommended pesticides for medicinal plants in the world [15] and this would probably explain the relatively high occurrence of the residues of these two OPPs in the collected samples. The amount of the OPPs residues in the plants depends on many factors including environmental, physicochemical properties of OPPs and biological properties of target plants. These factors may have synergistic or additive effects (negative or positive) on the half-lives of OPPs. Therefore it can be concluded that under some conditions, some of the OPPs may degrade or eliminate faster than the others from environment and be undetectable [16]. *Borage* had no detectable concentrations of any of the target OPPs. The probable explanation could be the insect repellant properties of *Borage* which significantly reduces the need for OPPs usage. It is noticeable that *Borage* is often cultured in vegetable gardens to protect other vegetables from insect damages [17]. By considering the cost benefit ratio and the widespread belief about organic products, the producers may prefer cultivation without OPPs in the case of *Borage*. From the viewpoints of pesticide fate on plant surface and in plant there could be another explanation for these results. Pesticide landing on plant surfaces may dissipate through volatilization, photolysis and by microbial activity on the leaf surface, as well as by washing off in rain or spray irrigation. Concentrations in or on plant tissue may also decline through growth dilution effects. Many pesticides adsorb to the leaf surface, move into the waxy surface of the cuticle or are absorbed into plant cells, reducing the amount of residues which might wash off and enabling degradation by plant enzymes to occur [18]. Due to the physiological, anatomical and biochemical properties of *Borage* the degradation processes might occur more intensive in *Borage* compared to the other studied plants. In a previous study the uptake and phytotransformation of OPPs was investigated in vitro using the axenically aquatic cultivated plants [19]. The study indicated that, the extent of decay and rate constants depended on both the physicochemical properties of the OPPs and the nature of the plant species. The results also showed that selected aquatic plants have the potential to accumulate and to metabolize OPPs; it also provided knowledge for potential use in phytoremediation processes [19]. *Borage* may uptake and transforms OPPs in a similar way. Consequently the concentrations of OPPs fall below detection limits of the analytical method. Therefore *Borage* may have a similar application in phytoremediation processes [20]. Many studies of pesticide residues in herbal materials have been carried out in other countries as well [15,21]. Previous studies showed the existence of malathion in spices and medicinal plants, with the highest mean level (2.19 mg kg^-1^) detected in *chamomile*[2,22,23]. In this study, the levels of malathion found in all samples are low and comparable with the results of other studies for fruits, vegetables and medicinal plants but much lower than the levels which are reported for medicinal plants collected from Egyptian markets which could be the result of training programs for framer held since 2002 in Iran [2,22-28]. Although not considerable, diazinon levels detected in some of the samples were higher than the ones asserted in the literature [2,22,23], However, the results revealed that the average amounts were still below the maximum residue levels (MRLs) proposed by the international organizations [11].

## Conclusions

A total of 50 samples of frequently used medicinal plants from different areas of Iran were analyzed for selected OPPs. At the detection limits of 0.5 ng g^-1^ parathion and pirimiphos methyl were not detected in any of the samples, whereas residues of diazinon and malathion were found in *Zataria*, *Matricaria chamomile*, *Spearmint* and *Cumin* Seed below the recommended MRLs. *Borage* had no detectable concentrations of any of the target OPPs which could be a result of intensive transformation of OPPs by *Borage.* Therefore *Borage* may have an application in phytoremediation processes.

## Competing interest

The authors declare that they have no competing interests.

## Authors’ contribution

P. Sarkhail (Plants provider and manager of plant sample preparation), M. Yunesian (Advisor and statistical analyst), R. Ahmadkhaniha (Chemical and GC-Mass analysis, prepartion of manuscript), P. Sarkheil (Asistant in plant sample preparation), N. Rastkari (Scientific director and developer of HS-SPME fiber and corresponding author). All authors read and approved the final manuscript.
